# Raman spectroscopy for medulloblastoma

**DOI:** 10.1007/s00381-018-3906-7

**Published:** 2018-07-12

**Authors:** Bartosz Polis, Anna Imiela, Lech Polis, Halina Abramczyk

**Affiliations:** 1 Department of Neurosurgery and Neurotraumatology, Polish Mother’s Memorial Hospital Research Institute, 281/289 Rzgowska St., 93-338 Lodz, Poland; 2 Laboratory of Laser Molecular Spectroscopy, Institute of Applied Radiation Chemistry, Faculty of Chemistry, Lodz University of Technology, Wroblewskiego 15, 93-590 Lodz, Poland

**Keywords:** Raman, Spectroscopy, Medulloblastoma, Embryonal tumor

## Abstract

**Purpose:**

The aim of the study is to use Raman spectroscopy to analyze the biochemical composition of medulloblastoma and normal tissues from the safety margin of the CNS and to find specific Raman biomarkers capable of differentiating between tumorous and normal tissues.

**Methods:**

The tissue samples consisted of medulloblastoma (grade IV) (*n* = 11). The tissues from the negative margins were used as normal controls. Raman images were generated by a confocal Raman microscope—WITec alpha 300 RSA.

**Results:**

Raman vibrational signatures can predict which tissue has tumorous biochemistry and can identify medulloblastoma. The Raman technique makes use of the fact that tumors contain large amounts of protein and far less lipids (fatty compounds), while healthy tissue is rich in both.

**Conclusion:**

The ability of Raman spectroscopy and imaging to detect medulloblastoma tumors fills the niche in diagnostics. These powerful analytical techniques are capable of monitoring tissue morphology and biochemistry. Our results demonstrate that RS can be used to discriminate between normal and medulloblastoma tissues.

Medulloblastoma (MB) is the most common embryonal tumor of the central nervous system (CNS) (70% of all). It is located only in the posterior fossa. In the group of patients < 18 years of age, it constitutes 18% of all brain tumors and 30% concerning the posterior fossa. The median of age is 9 years with two occurrence peaks, 2–4 and 6–8 years of age. The frequency of occurrence revolves around 0.5/100,000 children. A more frequent occurrence in the male sex is suggested (♂:♀, 1.8:1).

The basic feature of MB biology is its huge local malice and the ability to spread through cerebrospinal fluid (CSF). The contemporary comprehensive treatment of tumors consists of surgical removal followed by pharmacological chemotherapy and megavoltage CNS therapy. The principle is the use of surgical treatment at the first stage. Radiotherapy is not used in patients under 3 years of age who are treated exclusively with chemotherapy and palliative surgical procedure in primary disseminated disease (M2–M4 Chang classification).

The recurrence of the neoplastic process occurs most frequently during the first 2 years after the end of treatment. As a rule, it occurs at the place of the original location.

The results of the last years’ research quite unequivocally indicate the possibility of improving the effects of MB treatment with the application and appropriate selection of conventional methods. However, the maximum 5-year survival rates achieved at 75–85% for the standard-risk groups and 60–69% in the high-risk groups seem to be the limit.

Although precise delineation of the tumor excision border is a crucial step in patient treatment and survival, there are currently no methods able to differentiate normal tissue from tumor during operation. Traditional methods such as radiography, ultrasonography, computed tomography, and magnetic resonance imaging are insufficient in spatial resolution and have limited intraoperative availability [14, 16]. This could be counteracted by Raman spectroscopy, a promising simple, quick, and non-invasive method.

Raman spectroscopy (RS) and imaging (RI) are methods that measure inelastic scattering of light, providing information about vibrations of tissue components in samples. As a result, Raman spectroscopy can provide biochemical information of tissues without using any contrast agents [1, 3].

Recent years brought a great number of papers indicating usefulness of Raman spectroscopy in brain research on animals [11, 17, 23] as well as preliminary research on human brain [10, 11, 13, 15, 20]. However, the studies included individual cases. Here, we studied 11 cases of medulloblastoma and 3 samples from the safety margin as a control.

The aim of the study is to use Raman spectroscopy to analyze the biochemical composition of medulloblastoma and normal tissues from the safety margin of the CNS and to find specific Raman biomarkers capable of differentiating between tumorous and normal tissues.

## Materials and methods

### Study participants and tissue preparations

All experiments were performed in compliance with relevant laws and guidelines of the Bioethical Committee at the Polish Mother’s Memorial Hospital Research Institute in Lodz (53/216) and of the Ministry of Health of the Republic of Poland. Written informed consent was obtained from patients. The tissue samples consisted of medulloblastoma (grade IV) (*n* = 11). The tissues from the negative margins were used as normal controls.

Microtomed 16-μm-thick tissue sections were obtained from frozen blocks of the material removed during surgical operation at the Polish Mother’s Memorial Hospital (Lodz, Poland) and placed on CaF_2_ substrates (CRYSTAL GmbH, Germany) for Raman spectroscopy and Raman imaging measurements. Parallel 6-μm tissue sections were obtained and stained with H&E followed by histology examination for all the specimens by a certified neuropathologist from the Polish Mother’s Memorial Hospital Research Institute in Lodz. The tissue sections were examined by Raman spectroscopy and Raman imaging. MRI images were used for visualization and location of the tumor region for each patient.

### Raman spectroscopy and imaging

Raman images were generated by a confocal Raman microscope—WITec alpha 300 RSA (Ulm, Germany)—consisting of an Olympus microscope coupled with a 300-mm Czerny–Turner monochromator (Princeton Instruments Acton SP23000; 300-mm imaging triple-grating monochromator/spectrograph) and a thermoelectrically cooled CCD camera (ANDOR Newton DU970N-UVB-353; EMCCD chip with a 1600 × 200 pixel format, 16 μm dimension each) operating in the standard mode at − 64 °C with full vertical binning. The excitation laser beam was a second harmonic of the Nd:YAG laser (532 nm) which was focused on the sample with a × 40-magnification objective (NIKON CFI Plan Fluor C ELWD 40×: NA 0.60, WD 3.6–2.8 mm; DIC-M, C.C.0-2) to the laser spot of 1 μm determined by the laser wavelength and microscope objective being used. The average laser excitation power was 10 mW, with a collection (integration) time of 0.5 s and a spectral step of 2 cm^−1^ in the fingerprint range of 200–1800 cm^−1^ and high-frequency region of 1800–3600 cm^−1^. A piezoelectric table was used to record Raman images. The spectra were collected at one acquisition per pixel and a 1200-line-mm^−1^ diffraction grating with the spectral bandpass varying from about 5.5 cm^−1^ per pixel at about 200 cm^−1^ to about 3.3 cm^−1^ per pixel at 3600 cm^−1^. Raman images (50 × 50 μm, 100 × 100 points per line) from the fingerprint spectral regions of the human brain tissue from the tumor mass and from the safety margin were constructed.

Detailed methodology on data pre-processing and multivariate data analysis used in the paper is available elsewhere [6, 8, 9].

The percentage of blue and red areas of images presented in the “Results and discussion” section was calculated with Color threshold and Measure option of the ImageJ software (US National Institutes of Health, Bethesda, MD, USA).

## Results and discussion

Here we show results of the examination of 11 tissue samples of medulloblastoma compared with normal tissue from the safety margin.

**Fig. 1:**
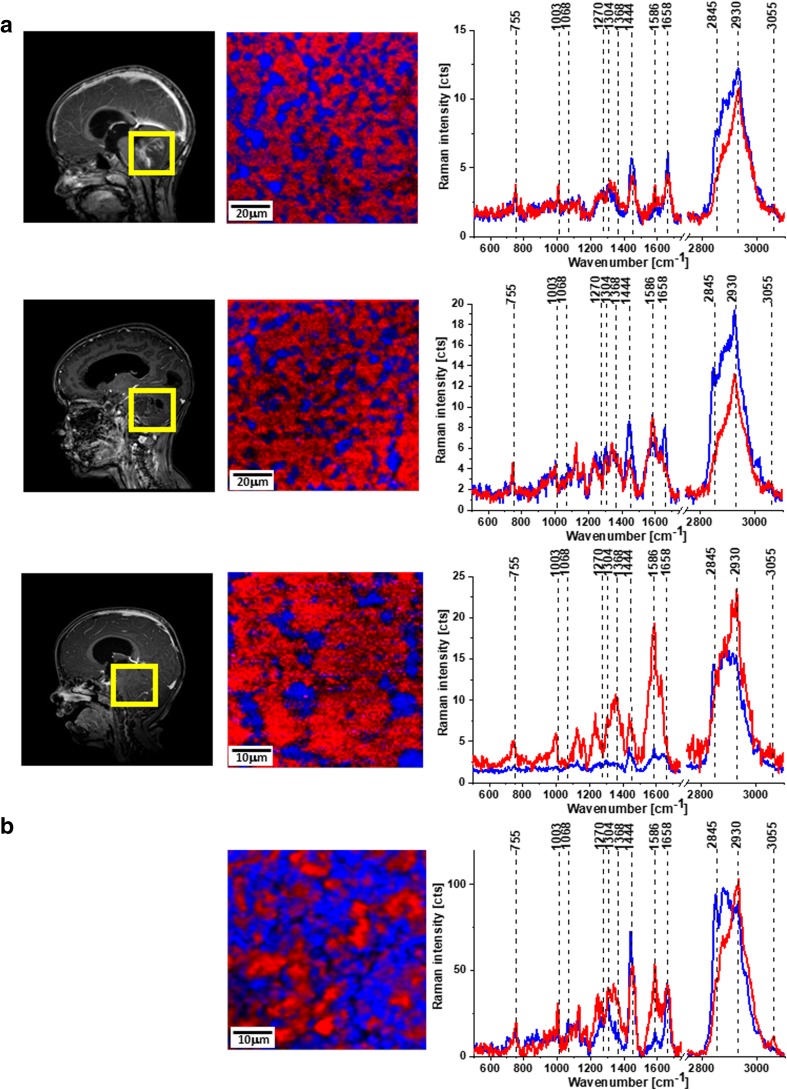
MRI images, Raman images, and Raman spectra of medulloblastoma (P27, P34, P38) (A) and Raman image and Raman spectra of normal tissue (B). The line colors of the spectra correspond to the colors of the Raman maps. Integration time for images, 0.5 s; resolution step, 0.5 μm; laser excitation power, 10 mW

Figure 1 shows MRI, microscopy, and Raman images as well as Raman spectra for medulloblastoma (A) and normal (B) tissues.

First of all, it is important to note that Raman mapping of human tissues can generate images as accurate as histology images with unique spatial resolution, sensitivity, and capabilities [1–4, 7].

A detailed insight into Fig. 1 shows spectral alterations in the chemical and morphological composition of the diagnosed medulloblastoma compared with normal tissue. The most important differences are in lipid and protein content marked on Raman images and spectra as blue (the band at 2845 cm^−1^ corresponds to CH_2_ sym. str. of lipids) and red (the band at 2930 cm^−1^ corresponds to CH_3_ sym. str. of lipids) areas, respectively.

The calculated areas of blue and red from Fig. 1 show 29, 24, and 20% of lipids for tumorous tissue and 58% area of lipids for normal tissue.

**Fig. 2:**
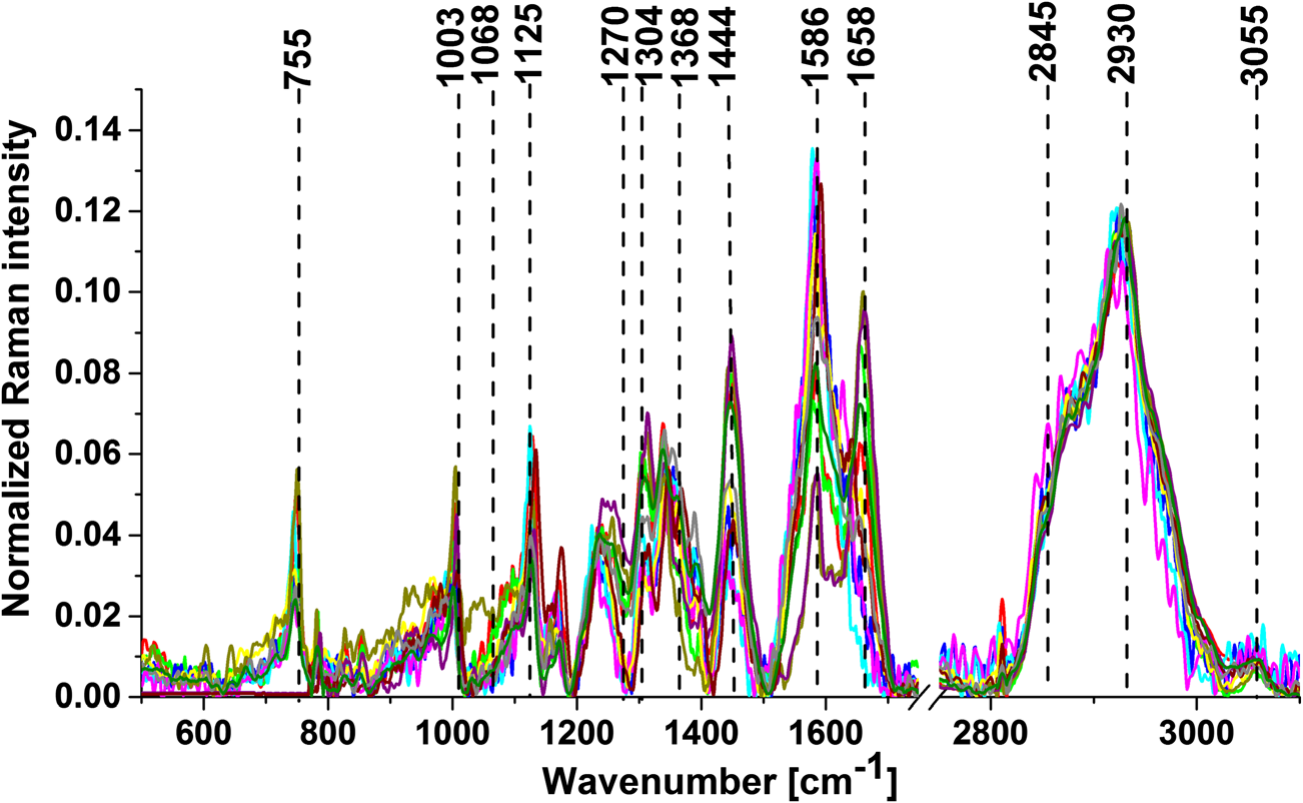
Average spectra from Raman images for 11 cases of medulloblastoma

Figure 2 shows vector-normalized average spectra from Raman imaging for all analyzed samples of medulloblastoma. The results show that the spectra of all samples were highly reproducible.

**Fig. 3:**
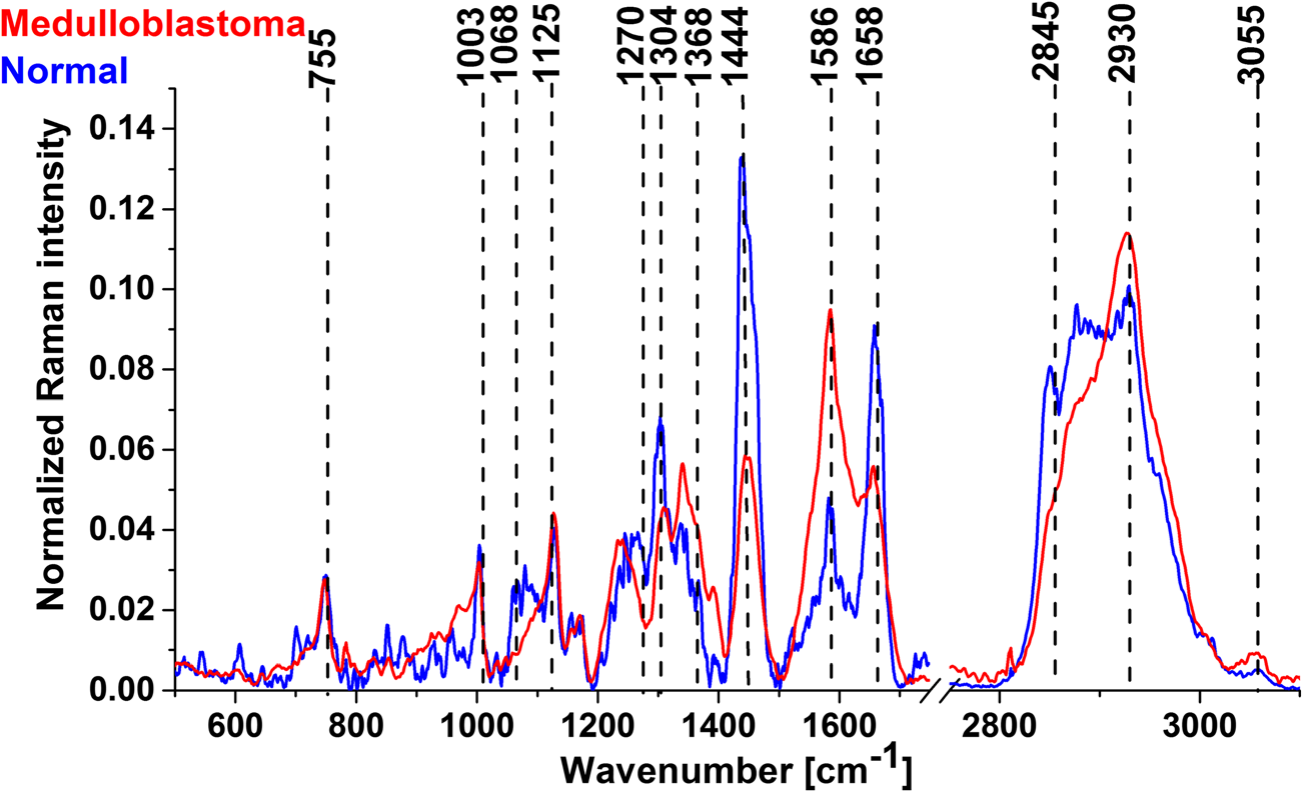
Average Raman spectra for medulloblastoma and normal tissues

Figure 3 shows the vector-normalized average spectra for medulloblastoma and normal tissues.

The results presented in Fig. 3 show that the Raman vibrational signatures can predict which tissue has tumorous biochemistry and can identify medulloblastoma. A detailed insight shows significant differences in lipid (1064, 1270, 1304, 1444, and 2845 cm^−1^) and protein (1240, 1368, 1586, 1658, and 2930 cm^−1^) content. Abramczyk et al. [1, 7, 26] showed that the Raman technique makes use of the fact that tumors contain large amounts of protein and far less lipids (fatty compounds), while healthy tissue is rich in both. Particularly interesting are the differences at 1586 cm^−1^, described as a marker of malignancy in tumors [21, 24]. Literature mostly merges this peak with the C=C bending mode of phenylalanine [5, 12, 19, 25], but our latest research shows that this also corresponds with phosphorylation of tyrosine. Also, the amide III band is shifted from 1270 to 1228 cm^−1^ as a result of phosphorylation. This is consistent with the latest research on phosphorylation inhibitors as a therapy for medulloblastoma [18].

For clinical application, the most important finding of the paper is the ratio of protein to lipid content presented in Table 1. This feature can be used to discriminate between normal and tumorous tissues. Table 1 shows the ratio for high frequencies and for the fingerprint region as well as the ratio calculated from the areas of proteins (*A*_proteins_) and lipids (*A*_lipids_) from Raman images.

**Table 1:** Raman intensity ratios at 2930/2845 cm^−1^ and 1586/1444 cm^−1^ for all analyzed medulloblastoma and normal samples

Medulloblastoma patient number	*I*_2930_/*I*_2845_	*I*_1586_/*I*_1444_	*A*_proteins_/*A*_lipids_
P3	2.74	1.30	1.85
P4	2.76	0.81	8.00
P9	3.2	3.81	3.34
P18	2.97	7.89	1.77
P27	1.90	4.64	4.00
P34	2.56	2.21	3.16
P38	3.25	0.56	2.44
P43	3.42	0.60	4.00
P44	2.86	2.90	2.70
P48	2.89	2.10	3.55
P49	3.17	1.13	6.69
Average for medulloblastoma	2.88 ± 0.26	2.54 ± 0.81	3.74 ± 1.24
Average for normal	1.6 ± 0.63	0.32 ± 0.18	0.77 ± 0.09

One can see that all studied cases of medulloblastoma tissue samples have the ratios *I*_2930_/*I*_2845_, *I*_1586_/*I*_1444_, and *A*_proteins_/*A*_lipids_ significantly higher than those for normal tissue, which demonstrates lower content of lipids in tumors. This is consistent with literature, where chromatography measurements show significantly reduced levels of polyunsaturated fatty acids (PUFA) and phospholipids in CNS tumors [22].

Both the ratios *I*_2930_/*I*_2845_ and *I*_1586_/*I*_1444_ can be used for diagnostic purposes. Although the results presented in the paper are highly reproducible, they are carried out on tissue slices, and for medical uses, more in vivo research should be conducted.

## Conclusions

The ability of Raman spectroscopy and imaging to detect medulloblastoma tumors fills the niche in diagnostics. These powerful analytical techniques are capable of monitoring tissue morphology and biochemistry. Our results demonstrate that RS can be used to discriminate between normal and medulloblastoma tissues by monitoring alterations in lipid and protein content using *I*_2930_/*I*_2845_ and *I*_1586_/*I*_1444_ ratios.
